# Integrated Metabolomics and Lipidomics Analysis Reveal Remodeling of Lipid Metabolism and Amino Acid Metabolism in Glucagon Receptor–Deficient Zebrafish

**DOI:** 10.3389/fcell.2020.605979

**Published:** 2021-01-14

**Authors:** Xuanxuan Bai, Jianxin Jia, Qi Kang, Yadong Fu, You Zhou, Yingbin Zhong, Chao Zhang, Mingyu Li

**Affiliations:** ^1^Fujian Provincial Key Laboratory of Innovative Drug Target Research, School of Pharmaceutical Sciences, Xiamen University, Xiamen, China; ^2^Translational Medical Center for Stem Cell Therapy and Institute for Regenerative Medicine, Shanghai East Hospital, Shanghai Key Laboratory of Signaling and Disease Research, Frontier Science Center for Stem Cell Research, School of Life Sciences and Technology, Tongji University, Shanghai, China; ^3^State Key Laboratory of Cellular Stress Biology, School of Life Sciences, Xiamen University, Xiamen, China; ^4^Center for Circadian Clocks, Soochow University, Suzhou, China; ^5^School of Biology and Basic Medical Sciences, Medical College, Soochow University, Suzhou, China; ^6^Division of Infection and Immunity, School of Medicine, Systems Immunity University Research Institute, Cardiff University, Cardiff, United Kingdom

**Keywords:** glucagon receptor, glucagon, metabolomics, lipidomics, zebrafish

## Abstract

The glucagon receptor (GCGR) is activated by glucagon and is essential for glucose, amino acid, and lipid metabolism of animals. GCGR blockade has been demonstrated to induce hypoglycemia, hyperaminoacidemia, hyperglucagonemia, decreased adiposity, hepatosteatosis, and pancreatic α cells hyperplasia in organisms. However, the mechanism of how GCGR regulates these physiological functions is not yet very clear. In our previous study, we revealed that GCGR regulated metabolic network at transcriptional level by RNA-seq using GCGR mutant zebrafish (*gcgr*^−/−^). Here, we further performed whole-organism metabolomics and lipidomics profiling on wild-type and *gcgr*^−/−^ zebrafish to study the changes of metabolites. We found 107 significantly different metabolites from metabolomics analysis and 87 significantly different lipids from lipidomics analysis. Chemical substance classification and pathway analysis integrated with transcriptomics data both revealed that amino acid metabolism and lipid metabolism were remodeled in *gcgr*-deficient zebrafish. Similar to other studies, our study showed that *gcgr*^−/−^ zebrafish exhibited decreased ureagenesis and impaired cholesterol metabolism. More interestingly, we found that the glycerophospholipid metabolism was disrupted, the arachidonic acid metabolism was up-regulated, and the tryptophan metabolism pathway was down-regulated in *gcgr*^−/−^ zebrafish. Based on the omics data, we further validated our findings by revealing that *gcgr*^−/−^ zebrafish exhibited dampened melatonin diel rhythmicity and increased locomotor activity. These global omics data provide us a better understanding about the role of GCGR in regulating metabolic network and new insight into GCGR physiological functions.

## Introduction

Glucagon is a 29-amino-acid polypeptide secreted by α cells from the islet of Langerhans, which is catalyzed from proglucagon by proconvertase 2 (Muller et al., 2017). Glucagon can specifically recognize and bind to the glucagon receptor (GCGR), which is widely distributed throughout the body and predominantly expressed in the liver (Burcelin et al., 1995; Muller et al., 2017). GCGR belongs to the class II G-protein–coupled receptor superfamily of seven transmembrane spanning receptors, which couples with GTP-binding proteins (G proteins) to adenyl cyclase, and activates the downstream signals to regulate glucose homeostasis through increasing glycogenolysis and gluconeogenesis (Wewer Albrechtsen, 2018; Qiao et al., 2020).

Studies have revealed that GCGR also plays many important roles in metabolism beyond glucose homeostasis (Charron and Vuguin, 2015; Galsgaard et al., 2019; Dean, 2020). Knockout or inhibition of GCGR in mice displayed pancreatic α-cell hyperplasia and increased the plasma concentrations of glucagon, glucagon-like peptide-1, low-density lipoprotein, and amino acids. Hyperaminoacidemia was observed in *Gcgr*^−/−^ mice in many studies (Yang et al., 2011; Solloway et al., 2015; Dean et al., 2017; Kim et al., 2017; Galsgaard et al., 2018; Winther-Sorensen et al., 2020). Moreover, a lot of genes involved in amino acid catabolism were found to be down-regulated in *Gcgr*^−/−^ mice, especially those related to glutamine, serine, and arginine metabolism (Yang et al., 2011; Dean et al., 2017; Kim et al., 2017; Winther-Sorensen et al., 2020). Further research revealed that deficiency of GCGR caused disturbed amino acid catabolism and reduced amino acid clearance in the liver and led to elevated plasma amino acid levels, which in turn stimulated the pancreatic α-cell hyperplasia and glucagon secretion (Dean et al., 2017; Galsgaard et al., 2018; Winther-Sorensen et al., 2020). These results suggested an endocrine loop of liver-α cell axis through glucagon signaling (Holst et al., 2017; Wewer Albrechtsen et al., 2018a,b; Dean, 2020).

Studies also showed that GCGR regulated the lipid metabolism through hepatic fatty acid β-oxidation and lipogenesis, and adipocytes lipolysis. Deficiency of GCGR led to decreased total body adipose mass and increased lean body mass, without changes in the total body weight (Gelling et al., 2003). After being fed with high-fat diet, the *Gcgr*^−/−^ mice had much smaller amounts of adipose tissue compared with wild type, suggesting they were resistant to diet-induced obesity (Conarello et al., 2007; Longuet et al., 2008). Nevertheless, *Gcgr*^−/−^ mice exhibited enhanced susceptibility to hepatosteatosis following exposure to the high-fat diet (Longuet et al., 2008). Similarly, knocking out GCGR and treatment with GCGR antagonist (LY2409021) induced liver fat accumulation in zebrafish and patients with type 2 diabetes (T2D), respectively (Guzman et al., 2017; Kang et al., 2020). Moreover, the plasma triglycerides and free fatty acids were increased in *Gcgr*^−/−^ mice after 16 h fasting (Longuet et al., 2008).

Metabolites are the final downstream products of cellular regulatory processes, which can powerfully reflect the physiological alteration in organisms. Metabolomics focuses on the identification of numerous metabolites (molecular weight <1,500 Dalton) and has a huge potential in characterizing physiological and biochemical activities (Fiehn, 2002). Lipidomics is a subset of metabolomics, which has similar functions to metabolomics but focuses on lipids (Lam et al., 2017). Although the serum metabolome has been studied in *Gcgr*^−/−^ mice (Yang et al., 2011; Dean et al., 2017), the whole-animal metabolomics changes are still unclear. In this study, we performed global metabolomics and lipidomics profiling using the whole organism of GCGR knockout zebrafish, which were further analyzed with our previous global transcriptomics data (Kang et al., 2020). We aimed to reveal the comprehensive metabolic alterations in *gcgr*^−/−^ zebrafish.

## Materials and Methods

### Zebrafish Husbandry

Zebrafish (*Danio rerio*) were raised in a recirculating aquaculture system (Shanghai Haisheng Biotech Co., Ltd, Shanghai, China) on a 14-h:10-h darkness cycle at 28°C. Embryos were obtained from natural breeding and raised at 28.5°C in embryo rearing solution and staged according to Kimmel et al. (1995). In this study, 7 dpf (days post fertilization) AB strain (wild type), *gcgra*^−/−^*; gcgrb*^−/−^ double-mutant fish (referred as *gcgr*^−/−^ henceforth) larvae were used. All procedures have been approved by the Xiamen University Institutional Animal Care and Use Committee (protocol XMULAC20160089, March 10, 2016).

### Sample Collection and Preparation for Global Metabolomics and Global Lipidomics Profiling

For metabolomics, 25 mg each of 7 dpf wild-type and *gcgr*^−/−^ mutant larvae were anesthetized and harvested into a 1.5 mL EP tube with 800 μL of precooled precipitant (methanol: acetonitrile: pure water = 2:2:1). Six biological repeats were performed for each group. After homogenization using ultrasonication, the samples were placed in the −20°C for 120 min to precipitate. Then the samples were centrifuged at 25,000 × *g* for 15 min at 4°C, and the supernatants were collected in new tubes for lyophilization in a centrifugal evaporator. After resupernation with 600 μL of 10% methanol solution, the samples were centrifuged at 25,000 × *g* for 15 min at 4°C, and then 5 μL of each sample was used for injection. The quality-control (QC) samples were mixed from 50 μL of each sample.

For lipidomics, 25 mg each of wild-type and *gcgr*^−/−^ mutant larvae added 800 μL of −20°C precooled dichloromethane/methanol (3:1) buffer solution. And the samples homogenized with TissueLyser for 5 min and then placed in at −20°C for 120 min. After the centrifugation and lyophilization, the samples were reconstituted with 600 μL of lipid complex solution (isopropanol–acetonitrile–water = 2:1:1). Finally, 5 μL of each sample was used for injection. The QC samples were mixed from equivalent amount of each sample.

### Ultraperformance Liquid Chromatography and Quadrupole Time-of-flight Mass Spectrum Condition

For metabolomics, ultraperformance liquid chromatography (UPLC) system (2777C, Waters, MA, USA) equipped with an ACQUITY UPLC HSS T3 column (100 × 2.1 mm, 1.8 μm, Waters, MA, USA) was used for separation. The column oven temperature was maintained at 50°C, and the flow rate was 0.4 mL/min. The mobile phase was consisted of solvent A (water + 0.1% formic acid) and solvent B (methanol + 0.1% formic acid). Metabolites were eluted using the following gradients: 0–2 min, 100% phase A; 2–11 min, 0% to 100% B; 11–13 min, 100% B; 13–15 min, 0% to 100% A. The small molecules eluted from column were detected by the high-resolution tandem mass spectrometer Xevo G2 XS QTOF (Waters, MA, USA) both in positive and negative ion modes. The capillary voltages were set at 3 kV (+) and 2 kV (–), respectively. The sampling cone voltage was set at 40 V in both modes. The Centroid MSE mode was used for data acquisition. The first-stage scan range of Time-of-flight mass was from 50 to 1,200 Da with the scan time of 0.2 s. For the tandem mass spectrometry (MS/MS) detection, the parent ions were fragmented using 20–40 eV with the scan time of 0.2 s. During the data acquisition, the LE signal was performed every 3 s to calibrate the mass accuracy.

For lipidomics, the separation was performed using UPLC equipped with an ACQUITY UPLC CSH C18 column (100 × 2.1 mm, 1.7 μm, Waters, MA, USA). The column oven was maintained at 55°C, and the flow rate was 0.4 mL/min. The mobile phase was consisted of solvent A (ACN: H2O = 60:40, 0.1% formate acid and 10 mM ammonium formate) and solvent B (IPA: ACN = 90:10, 0.1% formate acid and 10 mM ammonium formate). Gradient elution was performed in the following conditions: 0–2 min, 40–43% phase B; 2–7 min, 50–54% phase B; 7.1–13 min, 70–99% phase B; 13.1–15 min, 40% phase B. For the Q-TOF detection, the conditions were similar to metabolomics with some exception. The first-stage scan range of TOF mass was from 100 to 2,000 Da in positive mode and 50 to 2,000 Da in negative mode. For the MS/MS detection, the parent ions were fragmented using 19–45 eV.

### Data Processing and Analysis

For both global metabolomics and global lipidomics, the raw data obtained from the mass spectrometer were subjected to analysis by the Progenesis QI 2.2 (Waters, MA, USA), including peak alignment, peak picking, normalization, deconvolution, and peak identification. Then, the data were processed using metabolomics analysis R package metaX (BGI, Shenzhen, China) for further analysis. First, the low-weight ions with RSD (relative standard deviation) >30% were filtered and removed from the extracted data to ensure the metabolic quality. In addition, the data were corrected by QC-RLSC (QC-based robust LOESS signal correction) method (Dunn et al., 2011).

Next, in the univariate analysis, the fold change (FC) detection was performed. Simultaneously, the *t*-test analysis was used to calculate the *p*-value. In the multivariate analysis, principal component analysis (PCA) was performed for general clustering among the groups. Moreover, the partial least-squares discriminate analysis was employed to different ions between clusters, as influence intensity and explanation capacity of each ion were calculated by VIP (variable importance of projection), and components with VIP exceeding 1 were regarded as potential compounds contributing remarkably to the differences between groups. Based on results from QC sample detections, RSD threshold was set below 30% for error reduction, which was reflected in FC of metabolites. The metabolites identification was further performed through Human Metabolome Database (HMDB), Kyoto Encyclopedia of Genes and Genomes (KEGG), and LipidMaps. Finally, metabolites meeting the criteria (VIP > 1, *P* < 0.05, FC <0.83, and FC > 1.2) were selected as significantly different. All the raw data were deposited in the database of MetaboLights (www.ebi.ac.uk/metabolights/ MTBLS2067) (Haug et al., 2020).

Based on the different metabolites, we performed KEGG analysis by using R language especially the R package “clusterProfiler” (Yu et al., 2012). Further, integrative analyses of metabolomics and lipidomics with our previous transcriptomics data (Kang et al., 2020) were conducted through display of metabolites associated with genes on the pathways. On the basis of pathways analysis by different metabolites, the transcription level of relative genes in these pathways was selected for analysis. Criteria for different transcripts were set as follows: *Q* < 0.05, FC < 0.83, and FC > 1.2.

### Sampling of Zebrafish Larvae and Melatonin Enzyme-Linked Immunosorbent Assay

Seven-dpf zebrafish larvae were sampled every 4 h from ZT3 to ZT23 into tubes; each sample contained 20 larvae. After removing excess egg water, the tubes containing the samples were flash-frozen in liquid nitrogen. Samples were then stored at −80°C until use. This procedure was done in triplicate. Melatonin enzyme-linked immunosorbent assay (ELISA) assay was conducted according to the manufacturer's instruction (Zcibio technology Co., Ltd, Shanghai, China). Briefly, samples were thawed on ice, and 200 μL PBS was added to each sample. Samples were then homogenized and centrifuged at 12,000 rpm for 5 min at 4°C, and supernatants were used for ELISA. Fifty microliters of supernatant was pipetted into the melatonin antibody prepackaged 96-well microtiter plate, and then 50 μL of biotin antigen was added; the plate was covered and incubated at 37°C for 1 h. After incubation, the plate was washed three times, and 50 μL of enzyme conjugate was added and incubated for 30 min at 37°C, and then the plate was washed again for three times. Then, 100 μL of substrate solution was added, and the plate was again incubated for 15 min at 37°C. Finally, 50 μL of stop solution was added, and extinction was measured at 450 nm. The obtained optical densities of the standards (*y*-axis, logarithmic) were plotted against their concentrations (*x*-axis, logarithmic). A curve fit was performed, and the concentrations of the samples were then calculated from the standard curve.

### Zebrafish Behavioral Assays

Zebrafish larvae behavioral assays were performed under LD (light–dark) conditions as previously reported (Zhong et al., 2018). Briefly, a single zebrafish larva was placed in each well of 48-well plates at 7 dpf (24 WT, 24 *gcgr*^−/−^ mutant). The 48-well plate was placed inside the DanioVison system (Noldus, Wageningen, Holland), where white light was illuminated from 9:00 am to 11:00 pm (14 h light phase), and infrared light was set from 11:00 pm to 9:00 am (10-h dark phase). Locomotor activities of larvae were monitored for 2 consecutive days from 7 to 8 dpf using an automated video-tracking system, and the movement of each larva was recorded, and the average distance movement was analyzed.

## Results

### Global Metabolomics and Lipidomics Analysis of GCGR Knockout Zebrafish

**Figure 1:**
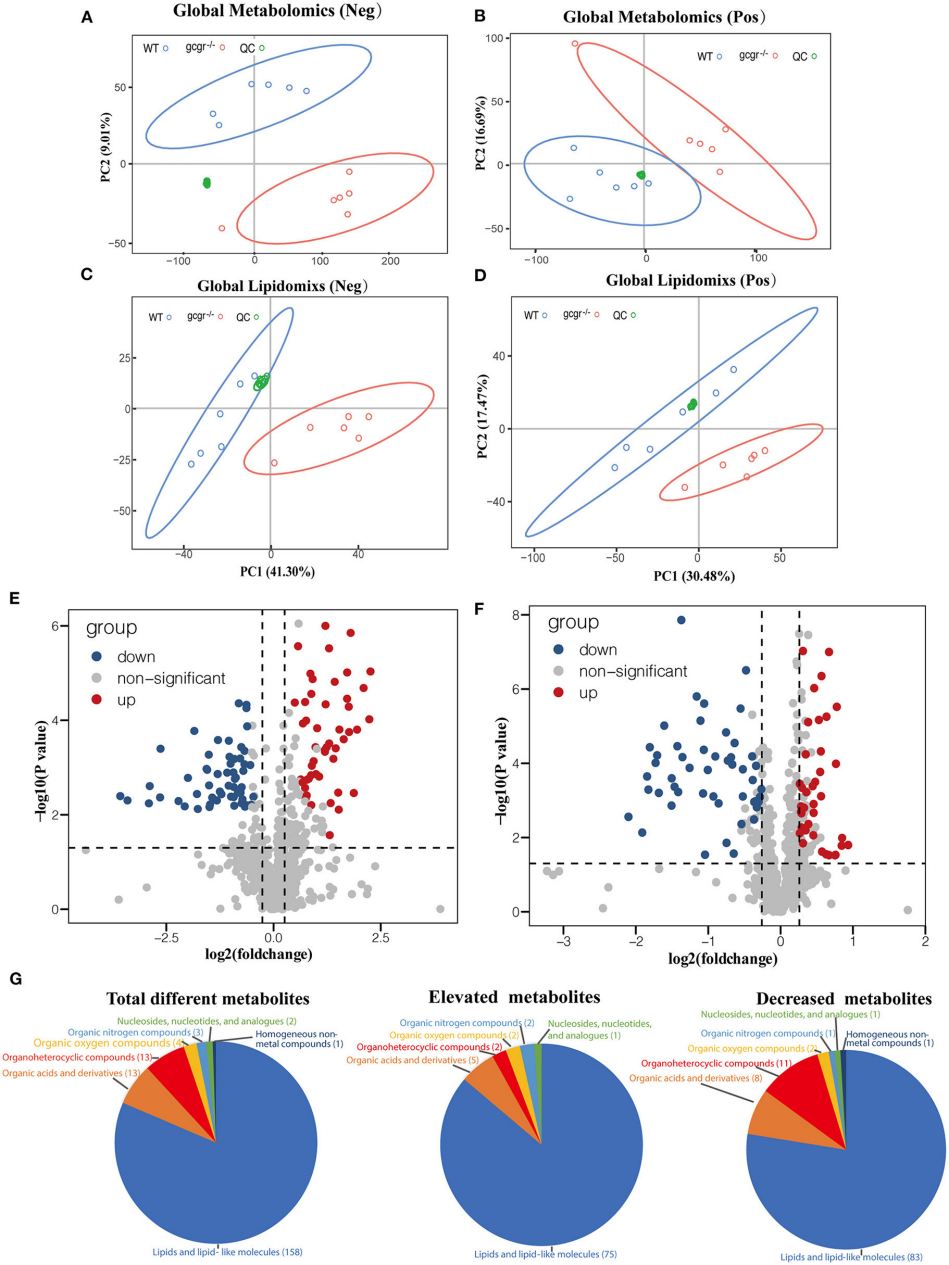
Profile of global metabolomics and lipidomics data. **(A–D)** Principal component analysis (PCA) of metabolomics **(A,B)** and lipidomics **(C,D)** profile of wild-type (WT) and *gcgr*^−/−^ were indicated by different color circles. PCA scores were plotted in both positive mode **(A,C)** and negative mode **(B,D)**. **(E,F)** Volcano plots of all identified metabolites from metabolomics **(E)** and lipidomics **(F)** analysis. The *x*-axis indicates Log2 (fold change) while the *y*-axis indicates -Log10 (*P*-value). Every single metabolite is represented as a dot. Different colors were used to represent down-regulated (blue), up-regulated (red), or non-significant (gray) metabolites. **(G)** Pie charts showing different classes of total different, elevated, and decreased metabolites. Different colors indicate different classes.

Global metabolomics and lipidomics were applied to profile the metabolic changes caused by GCGR deficiency in zebrafish. RSD threshold of 30% was displayed for 87.0% of metabolomics and 90.0% of lipidomics, respectively, indicating high reproducibility and stability. The profiles of QC groups, wild-type (WT) groups, and *gcgr*^−/−^ mutant groups were clustered separately in both negative and positive modes of the PCA plots (Figures 1A–D). These results indicated that the processing and analysis of HPLC-QTOF data met the required quantifications, and the differences between two experimental groups were significant. After filtration, 107 of 790 metabolites were identified to be significantly different by metabolomics analysis, with 49 up-regulated and 58 down-regulated in *gcgr*^−/−^ mutant groups (Figure 1E, Supplementary Table 1). Eighty-seven of 898 lipid molecules were identified to be significantly different by lipidomics analysis, with 38 up-regulated and 49 down-regulated in *gcgr*^−/−^ mutant groups (Figure 1F, Supplementary Table 1).

According to the HMDB database, the identified 194 different metabolites were matched to seven classes (Figure 1G, Supplementary Table 1). They were lipids and lipid-like molecules (number of metabolites, 158); organic acids and derivatives (13); organoheterocyclic compounds (13); organic oxygen compounds (4); organic nitrogen compounds (3); nucleosides, nucleotides, and analogs (2); and homogeneous non-metal compounds (1). Among these metabolites, the cluster of lipids and lipid-like molecules were the most abundant, with 75 increased and 83 decreased, indicating that the lipid metabolism was greatly influenced by GCGR deficiency.

### GCGR Knockout Induced Metabolic Disorder in Zebrafish

**Figure 2:**
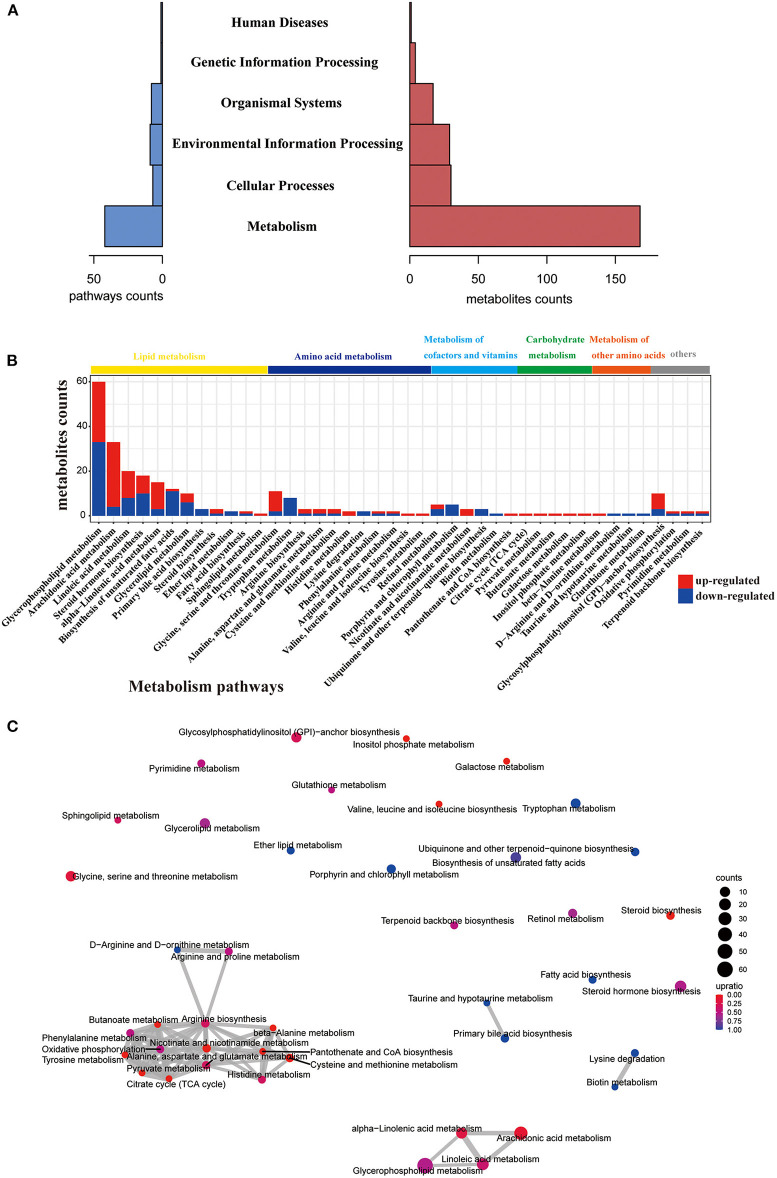
Pathway analysis of different metabolites. **(A)** Histogram of the holistic matching. All different metabolites are enriched into seven categories. The left blue bar indicates the number of enriched pathways. The right red bar indicates the number of covered metabolites. **(B)** Bar chart showing the number of metabolites covered by each enriched pathway. The *y*-axis indicates the number of metabolites while the *x*-axis indicates the pathway name. Red: increased; blue: decreased. The class of each pathway was labeled by the color bar on the top. **(C)** The network of enriched pathways. Each pathway is represented by a dot. Red: the ratio of the up-regulated metabolites is higher than down-regulated metabolites; blue: the ratio of down-regulated metabolites is higher than up-regulated metabolites. The dot size indicates the number of metabolites in the corresponding pathway. The line thickness represents the amount of substances shared by the linked pathways; the coarser, the more metabolites.

Next, we performed KEGG pathway analysis and pathway-based network analysis for these changed metabolites in *gcgr*^−/−^ mutant. The result showed that 176 different metabolites were enriched in 68 KEGG pathways, including metabolism (number of pathways, 42), environmental information processing (9), organismal systems (8), cellular processes (7), genetic information processing (1), and human diseases (1) (Figure 2A, Supplementary Table 2). Interestingly, metabolism-related pathways accounted for 61.8%% (42/68) of total enriched pathways covering 168 metabolites (Figure 2A, Supplementary Table 2). Thus, we then further analyzed these metabolism-related pathways in detail. The pathways related to lipid metabolism (number of pathways, 12), amino acid metabolism (11), metabolism of cofactors and vitamins (6), carbohydrate metabolism (5), and metabolism of other amino acids (4) were highly enriched (Figure 2C). Interestingly, 4 KEGG pathways containing more than 15 different metabolites were enriched, including glycerophospholipid metabolism (number of metabolites, 60), arachidonic acid (ARA) metabolism (33), linoleic acid metabolism (20), and steroid hormone biosynthesis (18), all belonging to lipid metabolism (Figure 2B and Supplementary Table 2). To explore the association and crosstalk between those metabolism-enriched pathways, the pathway-based network analysis was performed. As shown in Figure 2C, close linkages were shown in amino acid metabolism, carbohydrate metabolism, lipid metabolism, and so on, as several metabolites are shared by multiple pathways. Taken together, these data suggested that GCGR knockout in zebrafish induced metabolic disorder, especially in lipid metabolism and amino acid metabolism. The details regarding pathway perturbation were further discussed as follows.

### Lipid Metabolism Remodeling in *gcgr^−/−^* Mutant Zebrafish

**Figure 3:**
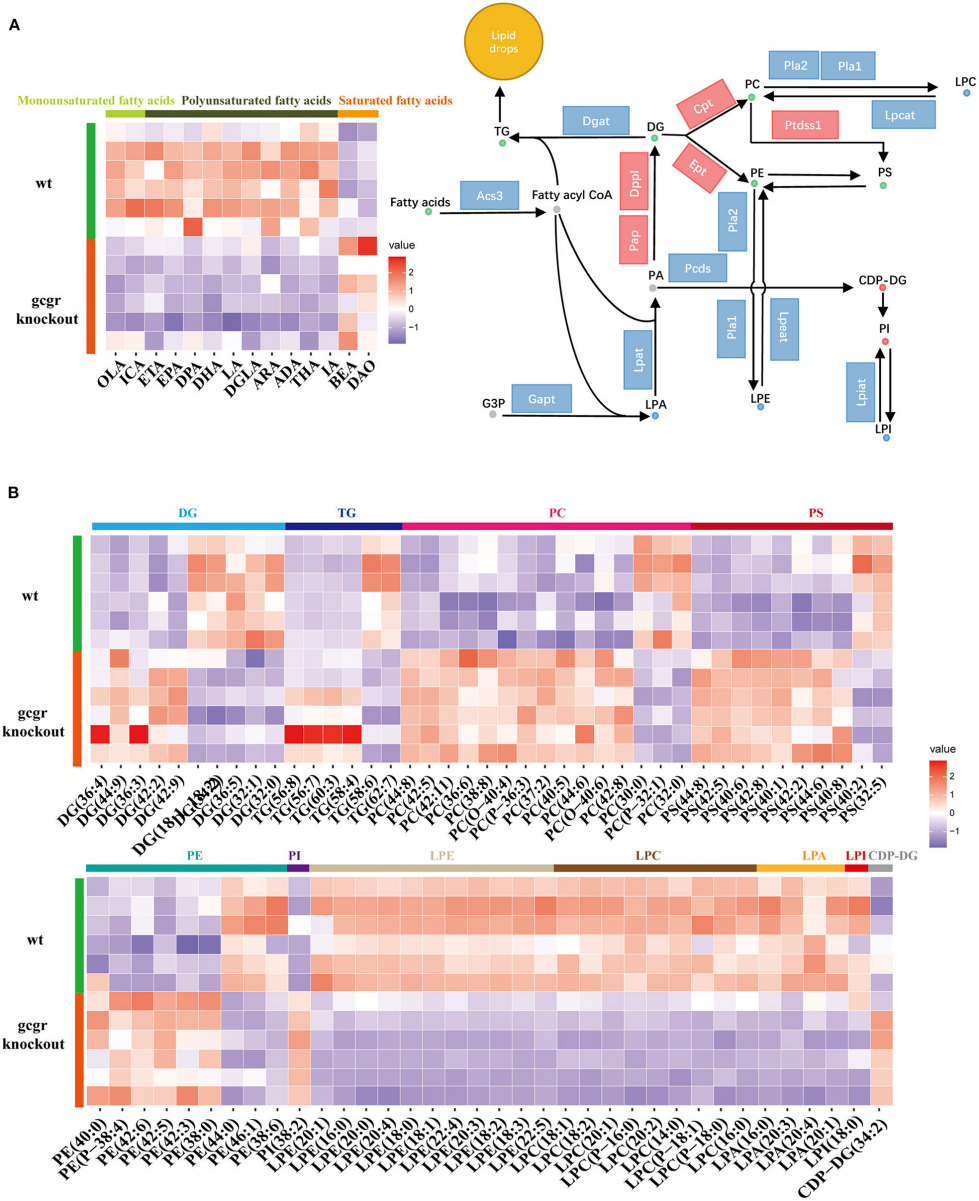
GCGR knockout induced glycerophospholipid metabolism dysregulation. **(A)** Disturbed glycerophospholipid metabolism. Dots represent metabolites, and blocks represent transcripts-encoded enzymes. Red: up-regulated, blue: down-regulated; or green: set substrates mixed with both up-regulated and down-regulated metabolites. The left displayed the heatmap enriched fatty acids. **(B)** Heatmap of corresponding glycerophospholipid metabolite sets. *Z* score normalized ionic strength for each metabolite was represented by different colors: high (red), low (blue), or average (white). The class of each pathway was marked by the color bar on the top.

Among these altered metabolism pathways, the lipid metabolism–related pathways were dramatically changed, which had 12 pathways enriched covering 125 metabolites enriched. Glycerophospholipid metabolism was the top enriched lipid metabolism pathway, with 60 significantly different metabolites, including classes of diacylglycerol (DG), triacylglycerol (TG), phosphatidylcholine (PC), phosphatidylserine (PS), phosphatidylethanolamine (PE), phosphatidylinositol (PI), lysophosphatidylethanolamine (LPE), lysophosphatidylcholine (LPC), lysophosphatidic acid (LPA), lysophosphatidylinositol (LPI), and cytidine diphosphate DG (CDP-DG) (Figures 3A,B, Supplementary Table 1). Fatty acids are utilized for production of fatty acyl-CoAs, which are essential component for glycerophospholipid metabolism (Watkins, 2013). Based on this, fatty acids were also analyzed here. Interestingly, the enriched monounsaturated fatty acids (oelsaeure and icosenoic acid) and polyunsaturated fatty acid [eicosatrienoic acid, eicosapentaenoic acid, docosapentaenoic acid, docosahexaenoic acid, linoleate, icosadienoic acid, dihomolinolenate, arachidonate (ARA), adrenic acid, tetracosahexaenoic acid] were all down-regulated (Figure 3A), whereas all the saturated fatty acids, including behenic acid and dodecanoic acid, were up-regulated (Figure 3A).

Moreover, all these LPEs, LPCs, LPAs, and LPI involved in glycerophospholipid metabolism pathways were significantly decreased, whereas CDP-DG and PI were increased in *gcgr*^−/−^ mutant (Figures 3A,B). Most of the PCs were increased after GCGR knockout, with only a few decreased. The same trend was observed for PSs and PEs. DGs were half increased and half decreased. Among six changed TGs, four of them (TG 56:8, TG 56:7, TG 60:3, TG 58:4) were up-regulated, whereas TG 58:6 and TG 62:7 were down-regulated. Among all of these changed lipids in glycerophospholipid metabolism, LPC (16:0) (−2.10) and LPC (P-18:0) (−1.91) were altered the most (Supplementary Table 1). To further investigate how GCGR affects lipid metabolism in multiple levels, we also carried out the integrated analysis of metabolomics data with our previous transcriptomics data (Kang et al., 2020). We found 29 significantly altered transcripts (Supplementary Table 3) were associated with glycerophospholipid metabolism. As shown in the Figure 3A, the transcriptional levels of genes encoded Pap, Dppl, Ept, Cpt, and Ptdsst1 were increased, whereas others such as Acs3, Lpat, Lpcat, Lpeat, and Lpiat were decreased, consistent with the metabolites profile (Figure 3A, Supplementary Table 3). Taken together, these data suggested glycerophospholipid metabolism reprogramming in GCGR-deficient zebrafish.

**Figure 4:**
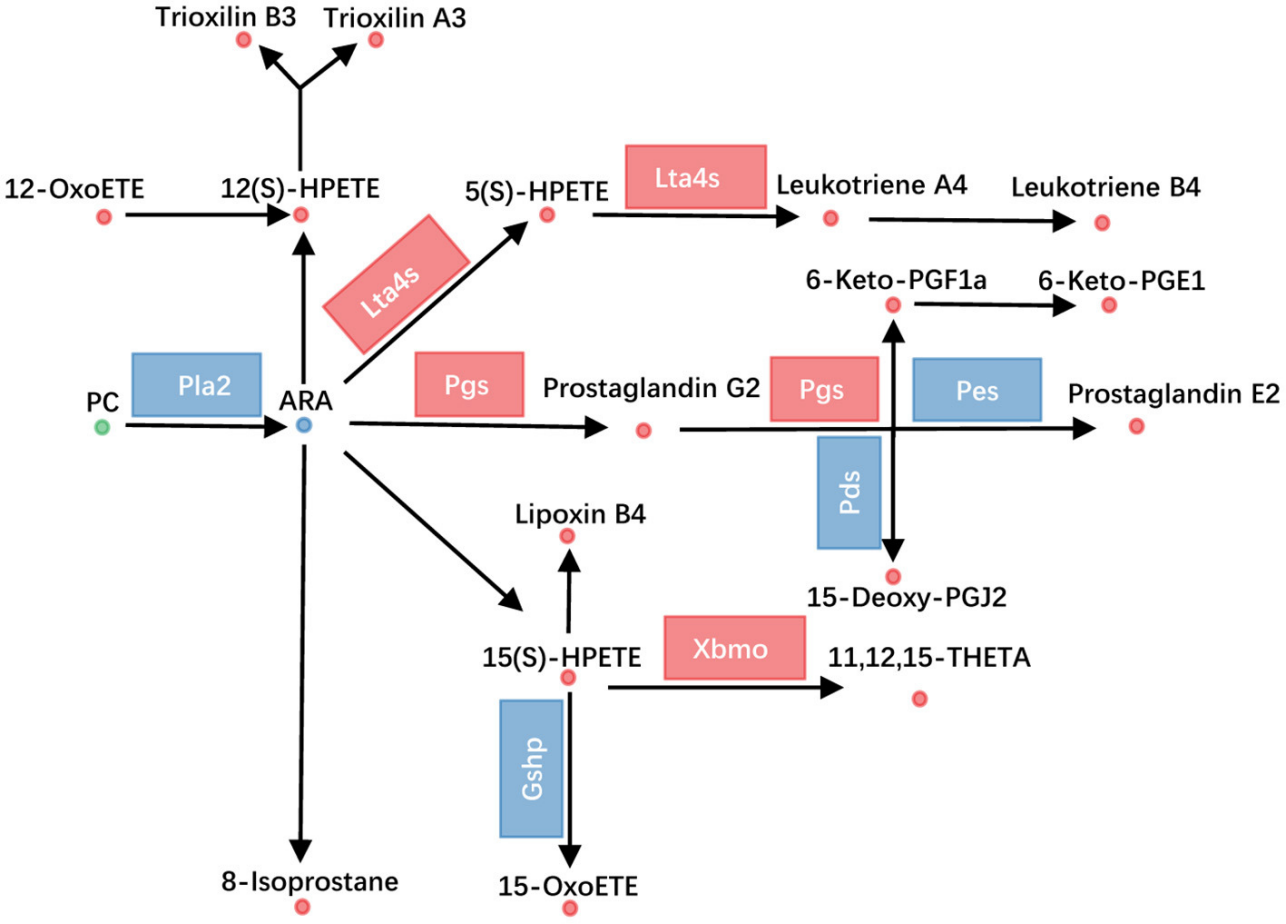
GCGR knockout influenced arachidonic acid metabolism. Disturbed arachidonic acid metabolism. Dots represent metabolites, and blocks represent transcripts-encode enzymes. Red: up-regulated, blue: down-regulated; or green: set substrates mixed with both up-regulated and down-regulated metabolites.

ARA metabolism was one of another dramatically changed pathway, with 33 metabolites altered. Fifteen PCs, which were the source for releasing of ARA, were altered (Figure 3B). Additionally, the transcriptional level of Pla2 (phospholipase A2) was decreased (Figure 4, Supplementary Table 3). ARA (ARA, −0.93) was down-regulated. Except for ARA and PC, all other 17 metabolites were increased (Figure 4, Supplementary Table 2). The products of ARA were eicosanoids, including prostaglandins, leukotrienes, lipoxins, thromboxanes, hepoxilins, isoprostanes, and hydroxyeicostetraenoic acids, which played important roles in organism physiology (Bend and Karmazyn, 1996; Sharma and Sharma, 1997; Fishbein et al., 2020). We found that the metabolites from six classes were increased, including prostaglandins consisting of PGE2 (prostaglandin E2, 0.93), PGG2 (prostaglandin G2, 1.20), 6-Keto-PGF1a (1.54), 6-keto-PGE1 (1.80), and 15-deoxy-PGJ2 (1.76); leukotrienes consisting of LTA4 (leukotriene A4, 1.87), LTB4 (leukotriene B4, 1.75), and 5(S)-HPETE (1.31); lipoxins consisting of LXB4 (lipoxin B4, 1.30), hepoxilins consisting of TXA3 (trioxilin A3, 1.43), and TXB3 (trioxilin B3, 1.21); hydroxyeicostetraenoic acids consisting of 15-OxoETE (2.23), 12(S)-HPETE (1.52), 15(S)-HPETE (1.64), 11,12,15-THETA (1.72), and 12-OxoETE (1.06); and isoprostanes consisting of 8-isoprostane (2.26) (Figure 4, Supplementary Table 2). Similarly, integrated analysis revealed transcriptional changes of related enzymes such as Lta4s, Pgs, and Xbmo, consistent with the alteration in metabolites (Figure 4, Supplementary Table 3). These data suggested that the ARA metabolism was up-regulated in *gcgr*^−/−^ mutant.

**Figure 5:**
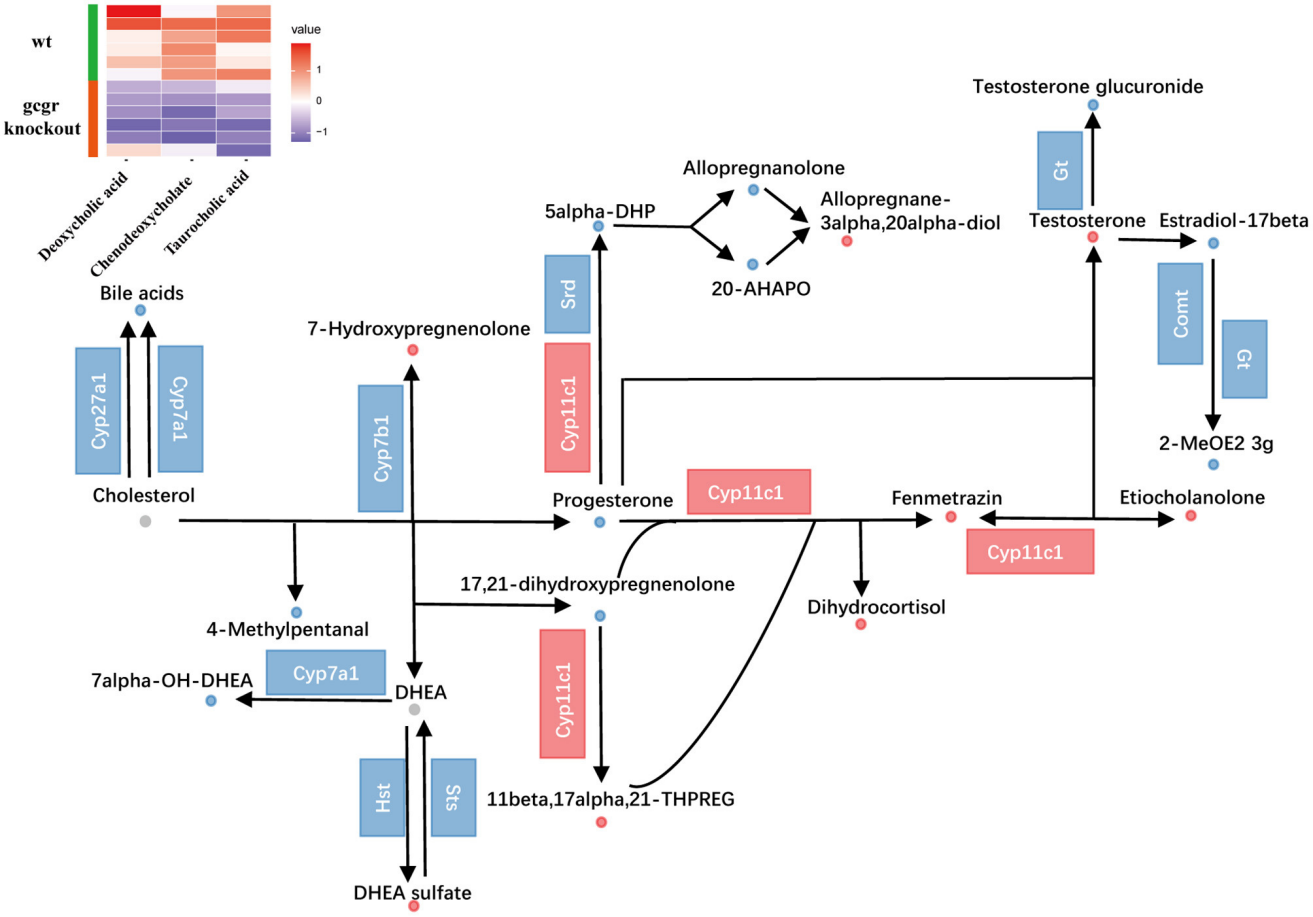
GCGR knockout influenced cholesterol metabolism. Dots represent metabolites, and blocks represent transcripts-encode enzymes. Red: up-regulated, blue: down-regulated; or green: set substrates mixed with both up-regulated and down-regulated metabolites. The heatmap indicates altered bile acids. *Z* score normalized ionic strength for each metabolite was represented by different colors: high (red), low (blue), or average (white).

Cholesterol metabolism was also influenced (Figure 5). The perturbed cholesterol metabolism was composed of steroid hormone biosynthesis pathway and biosynthesis of bile acid pathway. Through pathway analysis, steroid hormone biosynthesis was found to be dramatically changed with 18 altered metabolites (Supplementary Table 2). Among them, progesterone (−0.94) and testosterone (1.24) were significantly changed. As for bile acid metabolism, the mRNA levels of cholesterol 7alpha-monooxygenase (Cyp7a1) and sterol 27-hydroxylase (Cyp27a1) were decreased (Figure 5, Supplementary Table 3), which were important for bile acid synthesis. Moreover, the bile acids including taurocholic acid (−0.67), deoxycholic acid (−0.77), and chenodeoxycholate (−1.55) were all down-regulated, which were consistent with the alteration of related transcripts. These data suggested that the synthesis and content of bile acid in *gcgr*^−/−^ mutant zebrafish were decreased. Taken together, these data suggested reprogramming in cholesterol metabolism in GCGR-deficient zebrafish.

### Amino Acid Metabolism Remodeling in *gcgr^−/−^* Mutant Zebrafish

Previous studies have revealed that GCGR blockade decreased hepatic amino acid catabolism and increased the serum amino acid level, revealing the important role of GCGR in regulating amino acid metabolism (Solloway et al., 2015; Dean et al., 2017; Galsgaard et al., 2018; Winther-Sorensen et al., 2020). In our study, the KEGG pathway analysis also indicated that 11 pathways were enriched in amino acid metabolism, which was in the second place of metabolism-related pathways (Figure 2C).

**Figure 6:**
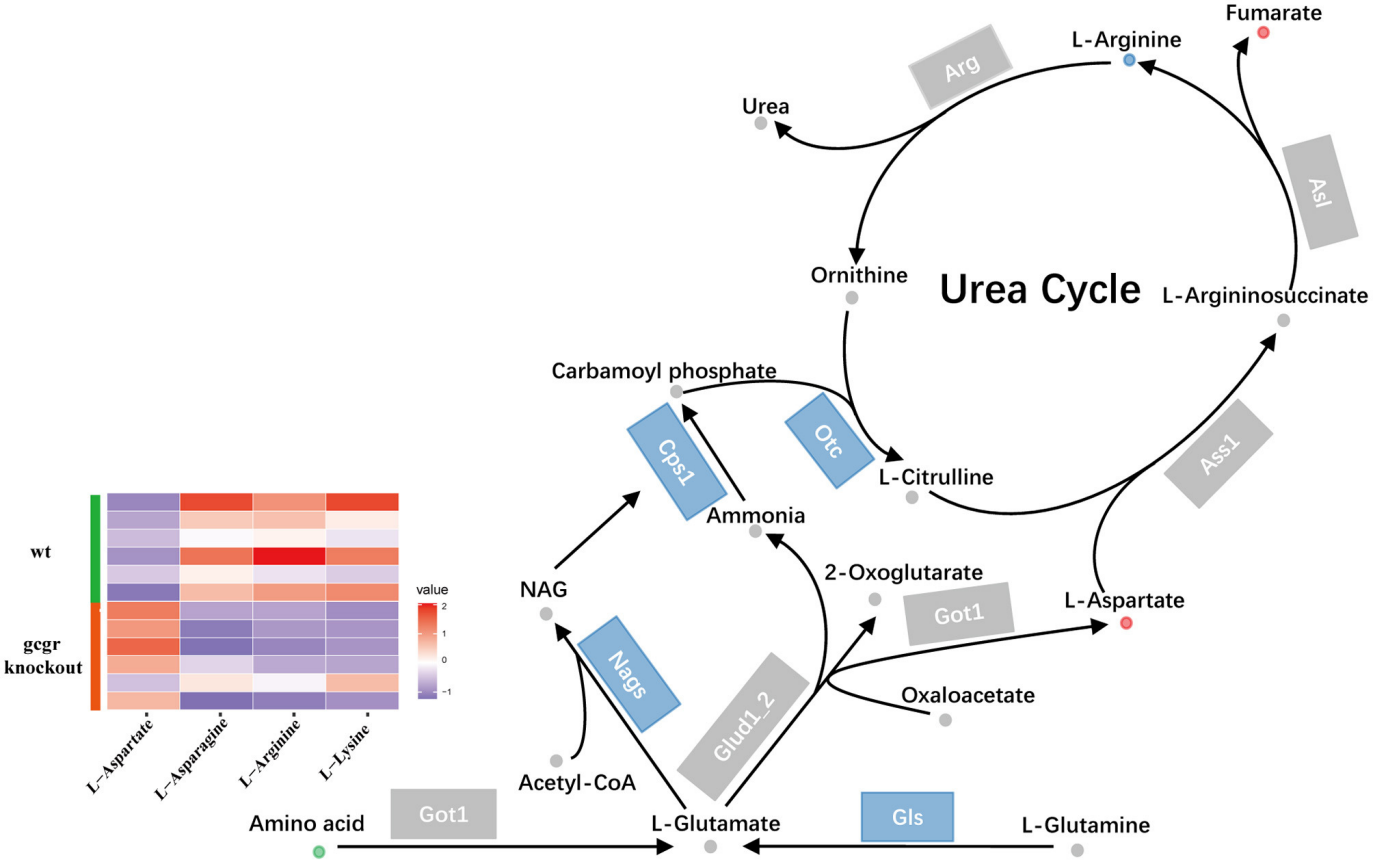
GCGR knockout influenced ureagenesis. Dots represent metabolites, and blocks represent transcripts-encode enzymes. Red: up-regulated, blue: down-regulated; or green: set substrates mixed with both up-regulated and down-regulated metabolites. The heatmap indicates altered amino acids. *Z* score normalized ionic strength for each metabolite was represented by different colors: high (red), low (blue), or average (white).

Glucagon pathway was one of the major regulators of ureagenesis (Morris, 2002). We also found that the ureagenesis included in amino acid catabolism was perturbed in *gcgr*^−/−^ mutant by analyzing the metabolomics and transcriptomics data (Figure 6). There were five significantly changed metabolites (Supplementary Table 1) and six significantly altered transcripts (Supplementary Table 3). Three metabolites were down-regulated, including l-lysine (−2.07), l-arginine (−2.00), and l-asparagine (−1.49), whereas l-aspartate (1.45) and fumarate (1.51) were increased. Several genes encoding the key enzymes involved in ureagenesis were significantly down-regulated, including Nags, Cps1, Otc, and Gls. Taken together, these data may suggest that ureagenesis was down-regulated in *gcgr*^−/−^ mutant.

**Figure 7:**
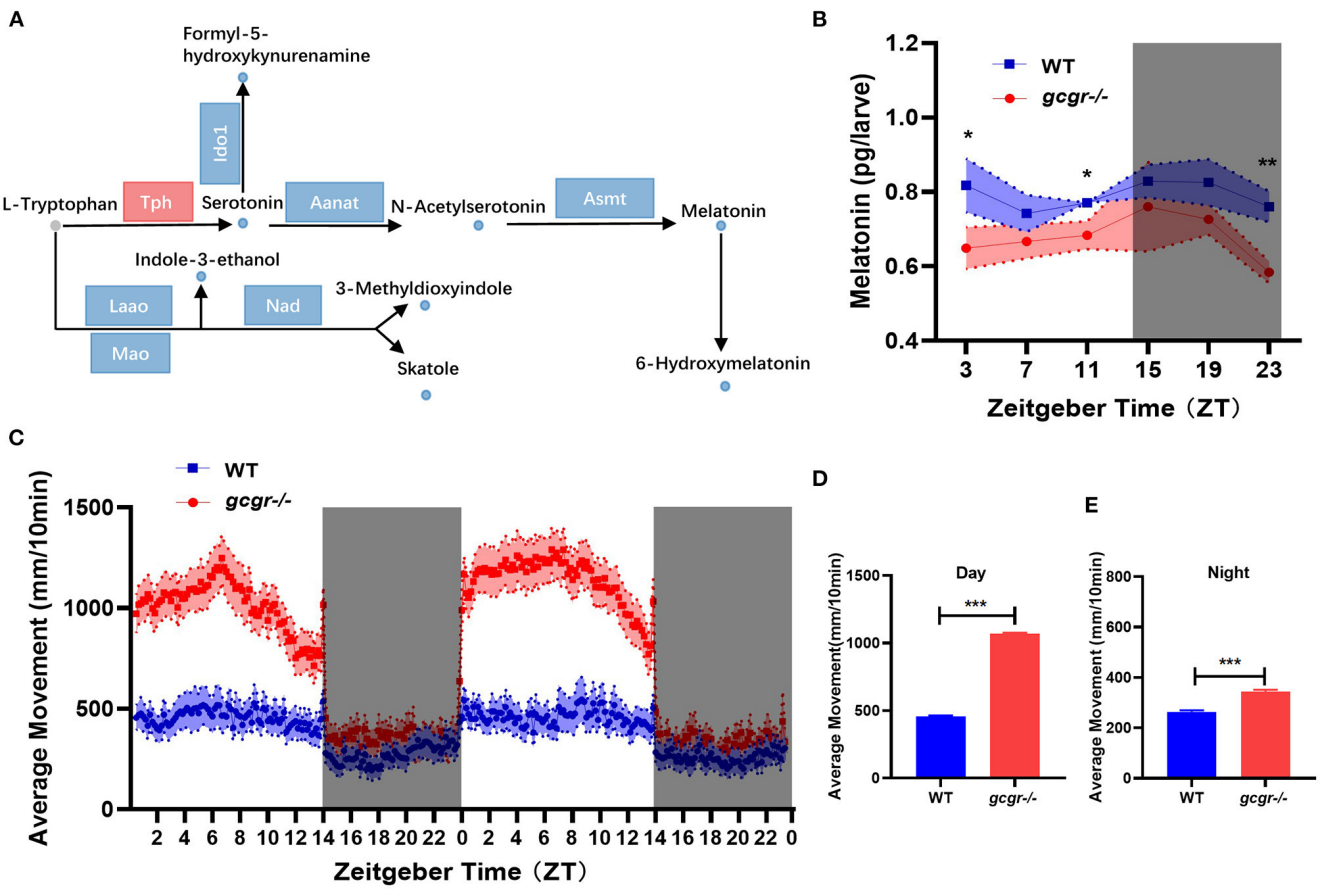
GCGR knockout influenced the tryptophan metabolism and locomotor activity in zebrafish. **(A)** Disturbed tryptophan metabolism. Dots represent metabolites, and blocks represent transcripts-encode enzymes. Red: up-regulated blue: down-regulated; gray: unvaried substrates. **(B)** Experimental validation of the melatonin level. **(C)** Locomotor activities were monitored in *gcgr*^−/−^ mutants and WT zebrafish larvae under LD condition. **(D,E)** *gcgr*^−/−^ mutants showed higher average moving distances at day **(D)** and night **(E)** than WT zebrafish larvae. Student *t*-test was conducted. *P < 0.05, **P < 0.01, ***P < 0.001.

Among the 11 enriched amino acid metabolism pathways, the tryptophan metabolism was dramatically affected in GCGR knockout zebrafish. Interestingly, all eight changed metabolites in the tryptophan metabolism pathway were decreased (Figure 7A). Notably, most metabolites involved in neurotransmitters serotonin and melatonin synthesis were significantly decreased, including serotonin (log2 FC, −1.77), n-acetylserotonin (−1.79), melatonin (−1.04), and 6-hydroxymelatonin (−1.85) (Figure 7A). Additionally, the mRNA levels of most enzymes participating in the tryptophan metabolism were significantly down-regulated, such as Ido1, Aanat, Asmt, and Nad (Figure 7A, Supplementary Table 3). Moreover, some other metabolites during tryptophan metabolism were also down-regulated, including indole-3-ethanol (−0.75), 3-methyldioxyindole (−1.00), skatole (−0.93), and formyl-5-hydroxykynurenamine (−1.25).

### *gcgr^−/−^* Mutant Zebrafish Dampened Melatonin Diel Rhythmicity and Increased Locomotor Activity

Melatonin is present in almost all organisms from bacteria to human, exerting autocrine and paracrine action and has been demonstrated to be expressed rhythmically, high at night and low during the day (Falcon et al., 2009; Cipolla-Neto and Amaral, 2018). Melatonin also plays an important role in the entrainment of daily and annual physiological and behavioral rhythms in vertebrates (Falcon et al., 2010; Pevet and Challet, 2011). Our metabolomics data suggested that melatonin was significantly decreased in *gcgr*^−/−^ mutant. We then further study the dynamic changes of melatonin and its influence on locomotor activity in *gcgr*^−/−^ mutant zebrafish. The melatonin level in *gcgr*^−/−^ zebrafish was higher than WT zebrafish in a whole cycle of Zeitgeber time (ZT), significantly in ZT3, ZT11, and ZT23 (Figure 7B). Correspondingly, *gcgr*^−/−^ zebrafish were more active, showing significantly enhanced moving distance (Figure 7C), both at day (Figure 7D) and night (Figure 7E), compared to WT zebrafish. These data suggested that GCGR knockout dampened melatonin diel rhythmicity and hence increased the locomotor activity in zebrafish.

## Discussion

Glucagon stimulates hepatic glucose output *via* activating GCGR in the liver. Antagonizing GCGR improves glycemic control in the diabetic state has been confirmed in clinic trials (Kazda et al., 2016; Scheen et al., 2017; Cheng et al., 2020). Nevertheless, blockade of GCGR by antagonists was accompanied by metabolic side effects, which could potentially limit the clinical application (Guan et al., 2015; Guzman et al., 2017; Scheen et al., 2017). Hence, a better understanding of the metabolic remodeling of organisms may provide useful information for antidiabetic therapies. In order to study the changes in GCGR-deficient animal, as well as for screening chemical modifiers to mitigate these side effects, we generated a GCGR knockout zebrafish model (Li et al., 2015). Our studies suggested that *gcgr*^−/−^ mutant zebrafish displayed phenotypes similar to *Gcgr*-deficient mice, which had lower free glucose content, higher glucagon content, α-cell hyperplasia, and accentuation of fat in the liver (Li et al., 2015; Kang et al., 2020). We further analyzed the regulated metabolic network in transcriptional level by RNA-seq and found that many genes related to metabolism of carbohydrates, lipids, and amino acids were dysregulated in *gcgr*^−/−^ mutant zebrafish (Kang et al., 2020). To get a more comprehensive understanding of *gcgr*-deficient zebrafish, we performed global metabolomics and lipidomics analysis of *gcgr*^−/−^ zebrafish and conducted integrated analysis combining metabolome, lipidome, and transcriptome in this study. We identified 107 different metabolites from metabolomics and 87 different metabolites from lipidomics analysis. We further revealed that many pathways related to lipid metabolism and amino acid metabolism were disrupted through pathway analysis.

GCGR signaling has been demonstrated to play important roles in the regulation of lipid metabolism in mammals (Charron and Vuguin, 2015; Galsgaard et al., 2019). Similarly, we also revealed that many pathways in lipid metabolism were disrupted at transcriptional level in *gcgr*-deficient zebrafish, which also displayed a significant lipid accumulation in the liver (Kang et al., 2020). Consistently, we revealed that cholesterol metabolism was impaired after GCGR knockout in this study (Figure 5). Besides, we found that glycerophospholipid metabolism and ARA metabolism were reprogramed in *gcgr*^−/−^ zebrafish.

Glycerophospholipids are the most abundant phospholipids, which are important lipid components in cellular membranes (Chauhan et al., 2016). In addition, glycerophospholipids are a source of physiologically active compounds that participate in the regulation of many cellular processes (Hishikawa et al., 2014; Rodriguez-Cuenca et al., 2017). The relationship between phospholipids metabolites and GCGR signaling has not been extensively studied. In our study, Most of PCs, PSs, and PEs were increased after GCGR knockout, and four of six changed TGs were also increased, whereas the amount of up-regulated DGs were equal to that of down-regulated DGs (5 vs. 5). Moreover, all the lyso-derivatives of glycerophospholipids, LPEs, LPCs, LPAs, and LPI, were down-regulated (Figures 3A,B). Consistently, the transcript level of lysophospholipid acyltransferases (Lpeat, Lpcat, Gapt, Lpiat) all declined (Figure 3A, Supplementary Table 3). Previous studies have shown that TGs and DGs were increased in obesity and T2D (Bitzur et al., 2009; Erion and Shulman, 2010; Markgraf et al., 2016), and PC levels were reduced in obese or insulin-resistant subjects (Razquin et al., 2018). Moreover, glucagon has been shown to stimulate hepatocyte TG secretion, and glucagon administration resulted in decreased TG plasma concentrations, as well as reduced hepatic TG content and secretion (Guettet et al., 1988; Bobe et al., 2003). However, how GCGR signaling regulates the glycerophospholipid metabolism remains to be further elucidated.

ARA metabolism was increased in GCGR knockout zebrafish (Figure 5), with 33 different metabolites involved in this pathway. Except for the ARA and PC set, all other 17 metabolites from this pathway were increased. These metabolites covering six classes of eicosanoids, including prostaglandins (PGG2, PGE2, 6-Keto-PGF1a, 6-keto-PGE1, 15-deoxy-PGJ2), leukotrienes (LTA4, LTB4, and 5(S)-HPETE), lipoxins (LXB4), hepoxilins (TXA3 and TXB3), hydroxyeicostetraenoic acids (15-OxoETE, 12(S)-HPETE, 15(S)-HPETE, 11,12,15-THETA, and 12-OxoETE), and isoprostanes (8-isoprostane). Metabolites derived from ARA metabolism have been implicated in immune surveillance, inflammation response, glucose metabolism, and lipid metabolism (Hartl and Wolfe, 1990; Tallima and El Ridi, 2018). And the metabolites of eicosanoids derived from ARA were involved in the regulation of pancreatic β-cell function, participating in the pathogenesis of diabetes and its complications (Luo and Wang, 2011; Sonnweber et al., 2018). Moreover, hyperglucagonemia caused by glucagonoma increased the levels of ARA, prostaglandins, and leukotrienes in patients (John and Schwartz, 2016). On the other hand, *in vitro* studies suggested that some of the prostaglandins can increase both basal and stimulated glucagon release (Giugliano et al., 1981; Walsh and Pek, 1984). However, how GCGR signaling participates in the regulation of ARA metabolism is yet to be explored.

GCGR blockade–induced hyperaminoacidemia has also been documented in mammals, from mice, monkey, to human patients (Okamoto et al., 2015; Larger et al., 2016; Galsgaard et al., 2018; Li et al., 2018). Studies further revealed that plasma hyperaminoacidemia was due to the decreased liver amino acid catabolism after GCGR inhibition (Solloway et al., 2015; Kim et al., 2017; Winther-Sorensen et al., 2020). High levels of plasma amino acids in turn stimulated the pancreatic α-cell hyperplasia, which has been defined as the liver-α cell axis (Dean et al., 2017; Galsgaard et al., 2018; Wewer Albrechtsen et al., 2018a). Similar to other studies, we found that the amino acid catabolism and ureagenesis were also downregulated in *gcgr*^−/−^ zebrafish (Figure 6) (Kang et al., 2020; and this study), and the *gcgr*^−/−^ zebrafish also displayed α-cell hyperplasia, suggesting that the liver-α cell axis is also conserved in zebrafish. Strikingly, several metabolites in the tryptophan metabolism pathway were dramatically down-regulated in *gcgr*^−/−^ zebrafish (Figure 7A). Tryptophan and its metabolites play many key roles in different physiological processes, including cell growth, immune response, neurotransmission, and enteroendocrine cell metabolism (Martin et al., 2019; Platten et al., 2019). Although the kynurenine pathway of tryptophan degradation did not change, the serotonin, melatonin, and three indole metabolites (indole-3-ethanol, 3-methyldioxyindole, and skatole) were significantly decreased. As melatonin functions as an important modulator of sleep and circadian regulation, as well as pancreatic hormone secretion (Yabut et al., 2019; Garaulet et al., 2020), we then measured the melatonin contents and locomotor activity in a whole cycle of ZT. Our results indicated that melatonin diel rhythmicity was dampened in *gcgr*^−/−^, which resulted in increased locomotor activity. Studies have demonstrated that increase in melatonin levels leads to enhanced α cells glucagon secretion both *in vitro* and *in vivo* (Ramracheya et al., 2008; Bahr et al., 2011, 2012). Moreover, knockouts of melatonin receptor 1 (MT1), melatonin receptor 2 (MT2), or both significantly elevated GCGR mRNA levels in the liver (Bahr et al., 2011). And pinealectomized diabetic rats (melatonin is predominantly secreted by the pineal) displayed an enhanced number of GCGRs and increased glucagon-binding activities in the liver (Mellado et al., 1989). All these data suggested that melatonin is highly associated with glucagon pathway. However, further studies need to be conducted to investigate the detail mechanism of how they interact and regulate the circadian rhythm.

**Figure 8:**
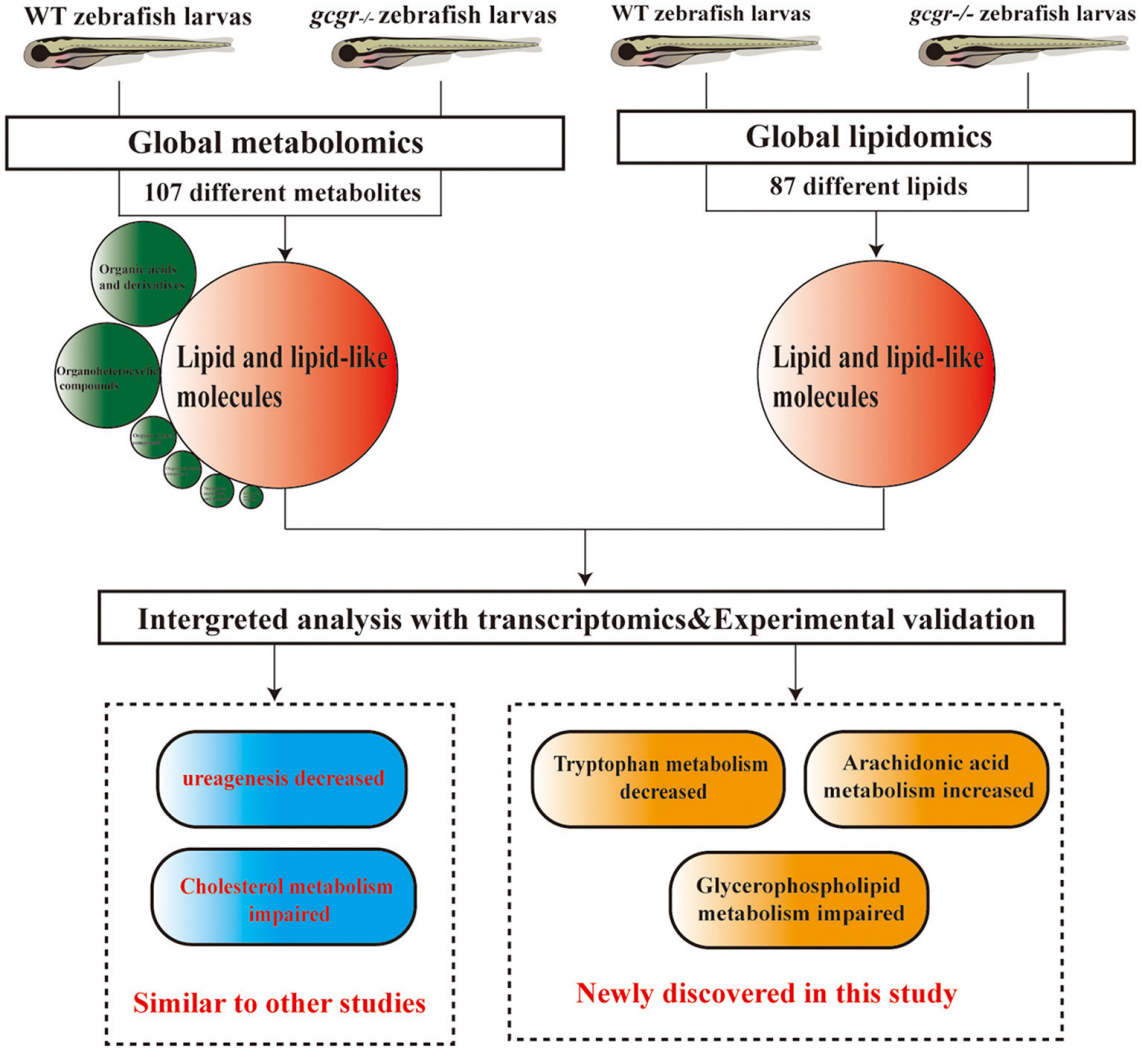
Summary of the experimental workflow and findings. Global metabolomics and lipidomics analysis were performed to study the metabolic change of *gcgr*^−/−^ mutant zebrafish. One hundred seven significantly different metabolites were found by metabolomics, and most of these metabolites were lipid and lipid-like molecules. Eighty-seven significantly different lipids were found by lipidomics. Integration analyses of metabolomics, lipidomics, and transcriptomics were then performed. Based on the analysis, we found that *gcgr*^−/−^ zebrafish displayed some similar metabolic changes to other studies. Importantly, we found that knockout of GCGR in zebrafish resulted in down-regulated tryptophan metabolism, up-regulated arachidonic acid metabolism, and disruption of glycerophospholipid metabolism.

In summary, we performed a global metabolomics and lipidomics study of *gcgr*^−/−^ mutant zebrafish, conducted the integrated analysis with global transcriptomics, and validated some findings in the model. We found that lipid metabolism and amino acid metabolism were remodeled in GCGR knockout. Consistent with GCGR studies in mammals, we also found that *gcgr*^−/−^ zebrafish showed decreased ureagenesis and impaired cholesterol metabolism. Beyond these, we also found that the glycerophospholipid metabolism was disrupted, the ARA metabolism was up-regulated, and the tryptophan metabolism pathway was down-regulated (Figure 8). These global omics data provide us a better understanding of GCGR in the metabolism remodeling and may provide useful information for GCGR antagonism therapies.

## Data Availability Statement

The original contributions presented in the study are included in the article/Supplementary Materials, further inquiries can be directed to the corresponding author/s.

## Ethics Statement

The animal study was reviewed and approved by Xiamen University Institutional Animal Care and Use Committee.

## Author Contributions

ML is the guarantor of this work and, as such, had full access to all of the data in the study and takes responsibility for the integrity of the data and the accuracy of the data analysis. ML, XB, QK, CZ, and YiZ designed the study. XB, JJ, QK, YF, and YiZ performed key experiments. ML, XB, QK, JJ, and CZ participated in the planning of the work and the interpretation of the results. XB drafted the manuscript. ML, QK, and YoZ revised the paper.

## Conflict of Interest

The authors declare that the research was conducted in the absence of any commercial or financial relationships that could be construed as a potential conflict of interest.

## Supplementary Material

Supplementary Table 1 Different metabolites in global metabolomics and lipidomics profiling.

Supplementary Table 2 KEGG pathway analysis of different metabolites.

Supplementary Table 3 Different transcripts corresponding to pathway analysis.
